# Nanostructures for Delivery of Flavonoids with Antibacterial Potential against *Klebsiella pneumoniae*

**DOI:** 10.3390/antibiotics13090844

**Published:** 2024-09-05

**Authors:** Hanne Lazla Rafael de Queiroz Macêdo, Lara Limeira de Oliveira, David Nattan de Oliveira, Karitas Farias Alves Lima, Isabella Macário Ferro Cavalcanti, Luís André de Almeida Campos

**Affiliations:** 1 Keizo Asami Institute (iLIKA), Federal University of Pernambuco (UFPE), Recife 50670-901, PE, Brazil; hanne.queiroz@ufpe.br (H.L.R.d.Q.M.); lara.limeira@ufpe.br (L.L.d.O.); david.dno@ufpe.br (D.N.d.O.); karitasfalima@gmail.com (K.F.A.L.); luis.andre@ufpe.br (L.A.d.A.C.); 2 Academic Center of Vitória (CAV), Federal University of Pernambuco (UFPE), Vitória de Santo Antão 50670-901, PE, Brazil

**Keywords:** natural products, nanotechnology, Gram-negative bacteria

## Abstract

Flavonoids are secondary metabolites that exhibit remarkable biological activities, including antimicrobial properties against *Klebsiella pneumoniae*, a pathogen responsible for several serious nosocomial infections. However, oral administration of these compounds faces considerable challenges, such as low bioavailability and chemical instability. Thus, the encapsulation of flavonoids in nanosystems emerges as a promising strategy to mitigate these limitations, offering protection against degradation; greater solubility; and, in some cases, controlled and targeted release. Different types of nanocarriers, such as polymeric nanoparticles, liposomes, and polymeric micelles, among others, have shown potential to increase the antimicrobial efficacy of flavonoids by reducing the therapeutic dose required and minimizing side effects. In addition, advances in nanotechnology enable co-encapsulation with other therapeutic agents and the development of systems responsive to more specific stimuli, optimizing treatment. In this context, the present article provides an updated review of the literature on flavonoids and the main nanocarriers used for delivering flavonoids with antibacterial properties against *Klebsiella pneumoniae.*

## 1. Introduction

*Klebsiella pneumoniae* is a Gram-negative bacterium belonging to the order Enterobacterales, commonly found in the human intestinal microbiota and frequently isolated from various environments, including aquatic bodies, food sources, and sewage systems [1,2,3]. *K. pneumoniae* can exist as symbionts, conditional pathogens, or outright pathogens, implicated in a range of infections such as urinary tract infections, liver abscesses, bacteremia, and pneumonia [4,5,6,7,8]. Notably, it is recognized as a nosocomial opportunist, particularly afflicting debilitated and immunocompromised patients. Its rapid dissemination within hospital settings underscores its significance as a hospital-acquired pathogen, contributing to considerable morbidity and mortality among hospitalized individuals [9,10].

In addition to these infections, *Klebsiella pneumoniae* is known for its capacity to develop resistance to antimicrobials, particularly exacerbated by the excessive and inappropriate use of antibiotics. The rising rates of bacterial resistance in *K. pneumoniae* present a global concern, as therapeutic regimens become less effective and the negative prognosis of patients increases significantly [3,11]. One of the primary antibiotic classes used to treat resistant strains of *K. pneumoniae* is carbapenems, given that a substantial proportion of resistant strains harbor a chromosomal β-lactamase gene, rendering penicillins such as ampicillin and amoxicillin ineffective [3,12]. However, there is currently a significant increase in the prevalence of carbapenem-resistant *K. pneumoniae* (CRKP) isolates, and infections caused by these strains are a global public health concern [13].

Given this situation, it is imperative to explore alternative therapies involving secondary metabolites with antibacterial potential, such as flavonoids. Flavonoids constitute a diverse group of polyphenolic secondary metabolites, characterized by their pigmentary properties, and are abundant in various foods, including coffee, fruits, and vegetables [14,15]. The term “flavonoids” derives from the Latin word “flavus”, meaning “yellow”. In plants, flavonoids play a crucial role in protecting against bacterial, fungal, and viral infections, oxidative damage, influencing fertility, regulating growth, and contributing to visual characteristics through their pigmentation [16,17,18,19,20]. The anti-inflammatory, antioxidant, and antimicrobial properties of flavonoids have been investigated in numerous experimental and clinical studies, demonstrating promising outcomes [14,21,22,23,24,25]. These characteristics underscore their value for further research, particularly in understanding their potential against bacterial, fungal, and viral infections.

These compounds are chemically characterized by two benzene rings, referred to as the A and B rings, linked by an oxygen-containing heterocyclic ring, known as the C ring. They can be categorized into various subclasses based on specific characteristics, including the type of linkage between the B and C rings; the molecular structure of the B ring; the distribution of hydroxyl and glycosyl groups across the three rings; and factors such as the level of saturation, oxidation, and hydroxylation in the C ring pattern. Consequently, these compounds can be classified into different subgroups, such as flavonols, flavones, flavanones, flavanols, isoflavones, chalcones, and anthocyanins [26]. This structural diversity contributes to a wide range of biological properties and potential health benefits associated with flavonoids, including anti-inflammatory, antioxidant, and antimicrobial activities [27,28].

Although flavonoids possess various pharmacological properties, their therapeutic application is hindered by several limitations, including photosensitivity, rapid metabolization, low bioavailability, and a short biological half-life. The low solubility in water is due to the presence of aromatic rings and hydroxyl groups (-OH), which form preferential interactions with each other, reducing interaction with water molecules. Photosensitivity results from the conjugated structure of flavonoids, which, upon absorbing light, can generate instability, free radicals, and photooxidation. Additionally, the rigid and planar structure, combined with multiple hydroxyl groups, hinders penetration through cellular membranes [29,30,31]. These characteristics have been identified as significant obstacles to their use as therapeutic agents. However, advances in nanotechnology offer the potential to overcome these challenges using nanocarriers for flavonoid delivery [32,33].

Nanosystems consist of particles or structures with nanometric dimensions, designed to achieve emergent properties at this scale, such as increased specific surface area and distinct optical, magnetic, and electronic characteristics. These systems are often synthesized to interact precisely and in a controlled manner with biological systems [34,35]. The size of nanoparticles is optimized according to the therapeutic target to maximize interaction with target cells and influence absorption mechanisms. Nanoparticles with extremely small dimensions may not be recommended in certain contexts due to their ability to traverse capillaries and reach unintended tissues or organs, which can result in adverse effects. In contrast, larger nanoparticles may have limitations in efficiently penetrating target cells, potentially reducing treatment efficacy. However, larger nanoparticles can be advantageous for specific applications, such as treatments targeting the intestinal tract [34,36,37,38].

## 2. Medicinal Plants: Prevalence, Synthesis, and Action of Flavonoids

Medicinal plants have played a significant role in human development and disease treatment. Historically, traditional communities have utilized plants to prevent and treat infections and various illnesses [39]. In this context, ethnopharmacology stands out, an area that studies the properties of bioactive natural compounds traditionally used by the population, thus being a source of discoveries of new safe and cheap medicines [40,41,42].

In this scenario, these natural compounds can be produced by a living being from its primary metabolism, used for growth and reproduction, or a secondary being, used as strategies for survival in stressful situations, for defense against pathogens and predators, and in resistance to climatic adversities [43]. There are studies showing the presence of flavonoids in some species of algae; however, the total amount of these compounds in these organisms is low. The presence of genes associated with the flavonoid biosynthetic pathway have also been studied in fungi. Despite this, outside the Plant Kingdom, the abundance of flavonoids in other living organisms is very low [44,45].

As products of secondary metabolism, flavonoids are part of the phytochemical profile of plants in great abundance and variety, depending on the taxonomic groups, since plants of the same family usually have a similar composition of metabolites, and on environmental conditions, such as the climatic conditions of the region, genotypes, and abundance of resources [46].

Flavonoids are primarily produced in plants through the phenylpropanoid pathway, which involves several enzymatic sub-pathways, including biosynthesis from phenylalanine. One of the flavonoids synthesized via this pathway is quercetin. The biosynthesis of quercetin begins with the conversion of phenylalanine into cinnamic acid by the enzyme phenylalanine ammonia-lyase (PAL). This is followed by the conversion of cinnamic acid into p-coumaric acid and subsequently into p-coumaryl-CoA. Chalcone synthase (CHS) then converts p-coumaryl-CoA into naringenin, which is hydroxylated to dihydroquercetin by flavanone 3-hydroxylase (F3H) and finally converted into quercetin by flavonol synthase (FLS) [24,47]. In 2020, Franco et al. [48] evaluated the phytochemical profile of *Bauhinia forficata* Link., an angiosperm of the Fabaceae family native to the Atlantic Forest and widely used in folk medicine, especially in the treatment of diabetes [49], and found 11 flavonoids including kaempferitrin, a flavonoid that mimics insulin. Another species whose phytochemical profile was analyzed is *Allium sativum* L., a member of the Amaryllidaceae family, known for its bulb used in both culinary and medicinal applications. The analysis revealed rutin as the predominant flavonoid, a molecule known for its various biological properties, including antimicrobial and anti-inflammatory activities [50,51,52]. Hamad et al. [51] extracted and purified rutin from *A. sativum* L., determining its immunomodulatory and anti-Schistosoma activity. Their findings indicated a reduction in hepatosplenomegaly and control of oxidative stress induced by the parasite. Abidullah et al. [53] demonstrated antibacterial activity against strains of *S. aureus*, *E. coli*, and *K. pneumoniae*, with inhibition zones measuring 28 mm, 27 mm, and 22 mm, respectively, for 10 μL quantities. Additionally, Wang et al. [54] demonstrated inhibition of biofilm formation and reduction in the expression of genes related to quorum sensing and bacterial virulence.

Another species whose phytochemical profile was analyzed is *Ginkgo biloba* L., the sole member of the Ginkgoaceae family of gymnosperms. Widely used in traditional Chinese medicine for treating bacterial and fungal infections, *Ginkgo biloba* is known for its high production of kaempferol, quercetin, and isorhamnetin, flavonoids responsible for its biological properties [55]. In this context, Chassagne et al. [56] demonstrated the plant’s efficacy in inhibiting bacterial growth and biofilm formation against *Streptococcus pyogenes*, *Staphylococcus aureus*, and *Cutibacterium acnes*, pathogens significant in “skin and soft tissue infections (SSTIs)”.

These compounds act as antioxidants and modulate polar auxin transport (PAT), thereby mitigating the effects of free radicals and enhancing plant robustness under adverse biotic and abiotic stress conditions [19,57,58,59]. Additionally, flavonoids contribute to the coloration and aroma of petals, promoting pollinator attraction [60,61]. In humans, flavonoids exhibit a broad spectrum of pharmacological properties, including antiviral, antiparasitic, antibacterial, glucose regulation, immune system modulation, neuroprotection, cardiovascular protection, rejuvenation, anticancer, and anti-inflammatory activities [23,60,62,63].

In addition to the antimicrobial activity exhibited by several families of medicinal plants due to their high flavonoid content, it has been found that these compounds, when used in conjunction with commercial antibiotics, can exhibit synergism and potentiate the drug’s effect. This synergy allows for dose reduction and decreased toxicity of conventional treatments, as well as aids in the combat of antimicrobial resistance (AMR) [52]. Among the flavonoids used in antibacterial combination therapy, quercetin is particularly noteworthy. Quercetin, a polyphenolic flavonoid, is a major component in various plant groups such as *Allium cepa* (onion), *Hypericum perforatum*, *Ginkgo biloba*, and *Camellia sinensis* [64].

**Figure 1:**
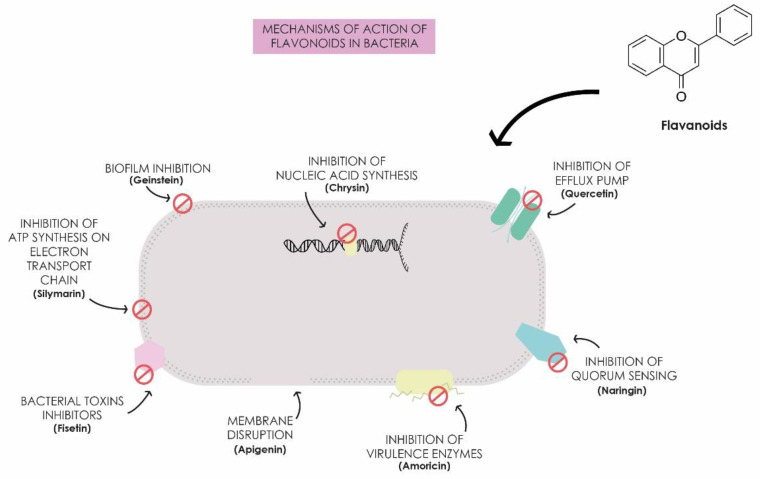
Mechanisms of action of flavonoids in bacteria.

Lin et al. [54] studied the effect of quercetin in combination with colistin to evaluate the reversal of colistin resistance in resistant *E. coli* and *K. pneumoniae* strains. The authors observed synergistic activity with fractional inhibitory concentration index (FICI), which consists of a mathematical method to assess whether the interaction between drugs is synergistic, additive, or antagonistic, with values less than or equal to 0.5 indicative of a synergistic effect. The obtained values ranged from 0.141 to 0.375, and 90% of the expression of the resistance gene *mcr-1* in *E. coli* and 95% of *mgr-B* in *K. pneumoniae* were inhibited. Pal and Tripathi [65] demonstrated the bactericidal and synergistic activity of quercetin in combination with meropenem against carbapenem-resistant *K. pneumoniae* and *E. coli*. They found synergistic activity with FICI values between 0.093 and 0.500 for *K. pneumoniae* strains, alongside altered expression of the *bla_VIM_* and *ompC* genes and changes in bacterial cell morphology. These studies underscore the potential of flavonoids as antimicrobial agents against clinically significant strains, highlighting their role in protecting plants against phytopathogens [27,66]. Several antibacterial mechanisms have been reported for these compounds, presenting activity through intracellular and extracellular mechanisms, including inhibition of nucleic acid synthesis, inhibition of bacterial motility, inhibition of the electron transport chain and ATP synthesis, inhibition of toxin production, inhibition of biofilm formation, inhibition of bacterial enzyme-dependent virulence mechanisms, membrane disruption, inhibition of efflux pumps, and prevention of quorum sensing (Figure 1). These compounds, therefore, not only interfere with the energy metabolism of bacteria but also inhibit the synthesis of nucleic acids, which are essential for bacterial replication and gene expression, effectively interrupting the life cycle of these microorganisms [67,68]. Thus, the use of plant-derived flavonoids represents a promising strategy for the development of new technologies in public health to combat antimicrobial resistance [21,69].

## 3. Nanocarriers Containing Flavonoids against *Klebsiella pneumoniae*

Flavonoids present an antibacterial therapeutic option, particularly for infections caused by antimicrobial-resistant strains of *Klebsiella pneumoniae*. However, their administration faces limitations, including low water solubility, which hampers absorption and bioavailability [70]. Furthermore, the chemical stability and pharmacokinetics of flavonoids can be compromised by metabolic processes, such as hepatic metabolism, intestinal degradation, and interactions with the intestinal microbiota, directly affecting their clinical efficacy. These factors constitute significant barriers to their application [71]. Consequently, loading these molecules into nanostructures emerges as a promising therapeutic approach for combating bacterial infections [15,72].

**Figure 2:**
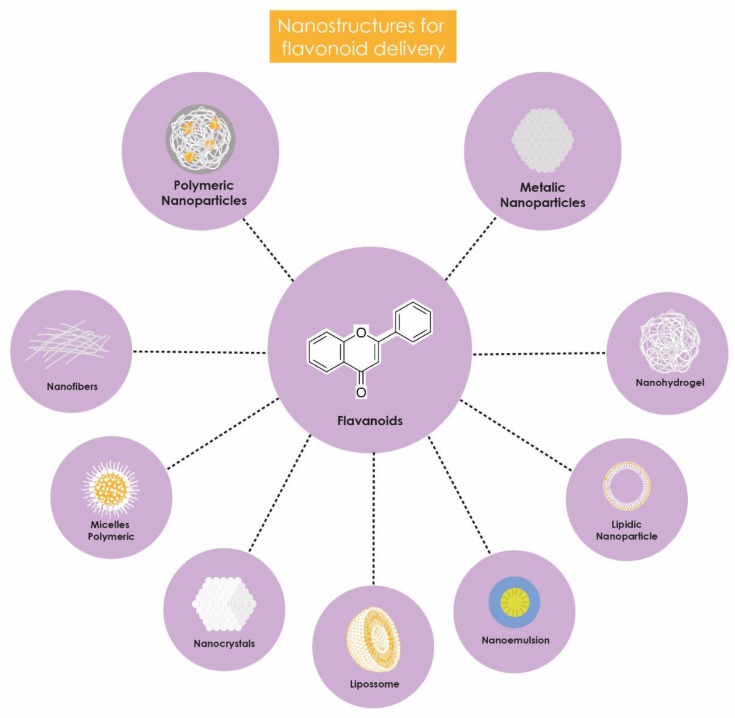
Benefits of flavonoid encapsulation.

These nanostructures enhance the bioavailability of flavonoids, improving their absorption and distribution within the body. Additionally, they offer protection against metabolic degradation, enable controlled release, and reduce side and adverse effects (Figure 2). Incorporation into nanocarriers also facilitates the penetration of flavonoids into target cells, optimizing their biological effects, particularly their antimicrobial action due to the targeted delivery of the encapsulated compound; the surface of the nanostructures can be functionalized with specific ligands or by altering charge or size, making the nanosystem have better affinity with bacterial cells and less impact on human cells, thereby reducing toxicity levels [73,74]. Consequently, numerous studies have evaluated the antimicrobial potential of nanotechnology-based formulations for delivering flavonoids against *K. pneumoniae* (Table 1).

### 3.1. Lipid Nanostructure

Lipid nanostructures are effective in encapsulating and releasing bioactive lipophilic compounds, utilizing lipid matrices to enhance solubility, stability, and bioavailability. Examples of such structures include nanoemulsions, nanostructured lipid carriers (NLC), and liposomes [58]. Nanoemulsions are colloidal systems composed of a mixture of two immiscible liquids, such as water and oil, stabilized by emulsifiers. These nanostructures are particularly suitable for topical administration due to their capability to penetrate the deeper layers of the epidermis [92].

Kaur et al. [75] developed nanoemulsions of catechins from *Camellia sinensis* to inhibit biofilm formation by resistant clinical isolates of *K. pneumoniae* collected from various health centers in Mumbai. The nanoemulsion was prepared using homogenization and ultrasonication techniques to combine bioactive compounds such as cranberry, curcumin, and Polyphenon 60. The antimicrobial activity of these nanoemulsions was evaluated using the microdilution method according to CLSI standards, and the antibiofilm activity was analyzed using the crystal violet method. The results indicated that growth was inhibited in 1:2 dilutions, containing 5.5 mg/mL of P60 and 15 mg/mL of cranberry (NE I), and 8 mg/mL of P60 and 2 mg/mL of curcumin (NE II). Additionally, there was significant efficacy in inhibiting biofilm growth, with an average inhibition of around 84% for the nanoemulsions, compared to approximately 64% for the free molecule.

In addition to nanoemulsions, nanostructured lipid carriers (NLCs) are utilized to deliver flavonoids. NLCs are drug delivery systems composed of a mixture of solid and liquid lipids that form a solid matrix. By increasing the available surface area, NLCs enhance the interaction of lipid fractions with epithelial membranes, facilitating adhesion to the walls of the gastrointestinal tract. Consequently, the formulation remains in the digestive system for a longer duration, thereby enhancing the absorption of the compounds [93,94,95].

Extracts rich in flavonoids are also effectively transported by liposomes, as demonstrated by Rubaka et al. [76]. They developed a liposomal nanocarrier loaded with *Carissa spinarum* polyphenols and coated with chitosan (Lip-CsP-chitosan) to enhance its antimicrobial action against *K. pneumoniae* ATCC. The antimicrobial activity was evaluated using the agar diffusion method. Lip-CsP-chitosan exhibited a relative inhibition zone diameter of 84.33 ± 2.51% compared to the free extract of *C. spinarum* and reduced the viability of *K. pneumoniae* by 57.45 ± 3.76% after 24 h, with a minimum inhibitory concentration of 31.25 mg/mL.

These studies demonstrate that the use of lipid nanostructures, such as nanoemulsions, NLCs, and liposomes, significantly enhances the delivery and efficacy of flavonoids against *Klebsiella pneumoniae*. These technologies are emerging as promising strategies to optimize the treatment of bacterial infections, providing an innovative approach to overcoming the limitations associated with the bioavailability and efficacy of antimicrobial agents.

### 3.2. Polymeric Nanostructures

Polymeric nanostructures are characterized by systems composed of polymers, which create nanostructured matrices for the controlled delivery of bioactive substances. These include nanogels, nanofibers, micelles, and polymeric nanoparticles. Nanogels, defined as polymeric matrices with dimensions in the nanometric scale, possess attributes that make them excellent carriers, including compatibility with biological systems, high encapsulation efficiency, precise release kinetics, and protection against degradation [96,97]. Based on these premises, Abbaszadeh et al. [77] developed a quercetin nanohydrogel using chitosan (ChiNH/Q). The antibacterial activity was demonstrated using the broth microdilution method, indicating the MIC against a resistant strain of *Klebsiella pneumoniae* PTCC 1290. This characteristic suggests its potential as an alternative antibiotic agent, as the nanohydrogel was able to reduce the MIC values for *K. pneumoniae* by 50%.

Chitosan is an abundant biopolymer widely used in nanotechnology for drug delivery systems due to its solubility, permeability enhancement, and mucoadhesive properties. It consists of a linear polysaccharide composed of D-glucosamine and N-acetyl-D-glucosamine units, linked by β-(1–4) glycosidic bonds, which allows for the formation of covalent bonds through reactions such as esterification, reductive amination, and etherification [98,99,100,101].

Abozahra et al. [83] investigated the antimicrobial activity of nanostructured lipid carriers (NLCs) containing rosemary (Baccharis dracunculifolia) and ginger (Zingiber officinale) essential oils, as well as the pharmaceutical form of chitosan nanoparticles against clinical isolates of *K. pneumoniae* obtained from patients at the Alexandria Fever Hospital [83]. Using the broth microdilution method, the minimum inhibitory concentration (MIC) of the essential oils was determined to be 1250 μg/mL. When encapsulated in NLCs, the MIC was reduced to 625 μg/mL. The lowest MIC was achieved with chitosan nanoparticles loaded with essential oils, enhancing the antibacterial efficacy of the essential oils against *K. pneumoniae* isolates by 8-fold. This positive effect may be attributed to the synergy between the essential oils and chitosan.

Nanofibers composed of biocompatible materials have frequently been utilized in therapeutic applications, proving to be excellent carriers for flavonoids. These structures are versatile, significantly enhancing patient adherence to therapy and yielding optimal results, particularly in wound healing applications [102,103,104]. In a study by Kannan et al. [78], electrospun nanofibers were synthesized containing flavonoid glycosides extracted from *Glinus oppositifolius*. These glycosides were previously isolated and subjected to a rigorous purification process using high-performance liquid chromatography methods and separation columns. The nanofibers demonstrated efficiency in antimicrobial tests, using the disk diffusion method against the resistant *K. pneumoniae* strain (MTCC 7028). The incorporation of flavonoid glycosides resulted in inhibition zones of 24 mm and 25 mm, exhibiting strong antibacterial activity

Polymeric micelles are spherical structures formed by surfactant polymers that self-organize in aqueous solutions. These micelles are excellent nanocarriers for antimicrobials, including flavonoids, due to their affinity with biological membranes and their ability to be incorporated into various pharmaceutical forms [105]. In a study by Miao et al. [79], methoxy poly(ethylene glycol)-poly(lactide) micelles loaded with luteolin (luteolin/MPEG-PLA) were formulated, demonstrating bactericidal effects against *K. pneumoniae.* The formulation not only increased the bioavailability of luteolin but also played a significant role in reducing inflammatory infiltrate in lung tissue in the murine models of lung infection induced by *K. pneumoniae*. These in vivo studies showed a significant therapeutic effect of luteolin/MPEG-PLA compared to free luteolin.

The encapsulation of flavonoids in polymeric nanoparticles, such as nanospheres and nanocapsules, enhances their controlled release and biological properties. Nanospheres are monolithic systems where the drug is dispersed or solubilized within a polymeric matrix, whereas nanocapsules consist of a polymeric membrane enclosing a core that can be solid or liquid. These nanocarriers improve the controlled delivery and efficacy of flavonoids at the target site. In this context, Balakrishnan et al. [80] developed poly(lactic-co-glycolic acid) (PLGA) nanoparticles loaded with hesperidin (HES-PLGA NPs). Their study, utilizing the broth microdilution method, demonstrated that HES-PLGA NPs at a concentration of 100 µg/mL exhibited significant antibacterial activity against multidrug-resistant clinical isolates of *K. pneumoniae*. Additionally, HES-PLGA NPs showed superior antioxidant activity compared to the reference standard ascorbic acid.

Alotaibi et al. [81] conducted a study involving the synthesis of PLGA nanocapsules loaded with epigallocatechin gallate (EGCG PLGA NCs). The antibacterial efficacy of free EGCG against the resistant strain *K. pneumoniae* ATCC 13883 was assessed using the well plate method, revealing an MIC of 128 µg/mL. In contrast, the MIC for EGCG encapsulated within PLGA nanocapsules was significantly lower at 16 µg/mL. Additionally, these nanocapsules demonstrated potential in mitigating kidney damage induced by cisplatin in murine models. This protective effect was evidenced by a notable reduction in renal biomarkers, including serum creatinine, KIM-1, and NGAL, alongside improvements in histopathological alterations associated with cisplatin-induced nephrotoxicity [106].

PLGA is widely utilized as a biopolymer for the synthesis of nanostructures due to its high performance and advantageous characteristics for drug delivery, including its ability to facilitate intracellular hydrolysis [95]. The encapsulation of isolated compounds and plant essential oils in PLGA has garnered significant scientific interest, particularly because these substances often contain high levels of flavonoids, which are critical to their therapeutic efficacy [107].

Zhang et al. [70] investigated the antimicrobial properties of essential oils extracted from guava leaves (*Psidium guajava*) when incorporated into chitosan nanoparticles targeting an environmental strain of *K. pneumoniae*. The study employed the ionic gelation technique, where the essential oil was loaded onto the surface of chitosan nanoparticles, with visual evidence of a bulge within the chitosan matrix, surrounded by discrete particles, as detailed by Hadidi et al. [108]. The guava leaf extract exhibited an inhibition zone of 12 mm against the multidrug-resistant *K. pneumoniae* strain. When loaded onto chitosan nanoparticles, the essential oil produced an inhibition zone of 22 mm at a concentration of 70 µg/mL.

In another study, Qanash et al. [82] investigated the encapsulation of *Artemisia judaica* extract, known for its high flavonoid content, into chitosan nanoparticles (CNPsLE) and assessed its antibacterial efficacy against clinical isolates of *K. pneumoniae* sourced from the Egyptian University Hospital using the broth microdilution method. High-performance liquid chromatography (HPLC) analysis of the *Artemisia judaica* (aerial parts) revealed the presence of 18 known compounds, with kaempferol, a natural flavonol, identified as the most predominant. Additionally, the extract contained other flavonoids such as luteolin, apigenin, rutin, and quercetin. Notably, the MIC for *K. pneumoniae* decreased approximately 7.62-fold when the extract was encapsulated in CNPsLE, with the MIC value of the free extract being 31.25 µg/mL compared to 4.1 µg/mL for the encapsulated form.

### 3.3. Metallic Nanostructure

Metallic nanostructures are used in various applications including, dentistry, molecular biology, cancer therapy and antimicrobial treatment [109,110,111]. The metallic nanostructures can be categorized in four types depending on the metal used in the synthesis process and on the physicochemical properties analyzed in the characterization method that those particles are based on: carbon, inorganic structures, organic structures, and composites [112].

Nanocarriers for flavonoid delivery have been extensively studied, with significant research focusing on their effectiveness, particularly when combined with gold nanocomposites, porous CuO nanobastons, magnetic iron oxide nanoparticles, and silver nanoparticles. Alhadrami et al. [84] developed gold nanocomposites (GNPs) coated with flavonoids, including quercetin, kaempferol, and chrysin, using glutathione (GSH) as a ligand to facilitate the interaction between the gold nanoparticles and the flavonoids. The antibacterial activity of these nanoconjugates was assessed using the agar diffusion method against the resistant strain *K. pneumoniae* (ATCC BAA-1705). The findings revealed that GNP-quercetin demonstrated the highest antimicrobial activity, with an MIC of 60 μg/mL. The authors proposed that these nanoconjugates may exert their antibacterial effects through two mechanisms: enhancing the rigidity of the bacterial membrane and inhibiting the B subunit of DNA gyrase (Gyr-B). Both in silico and in vitro analyses supported quercetin’s efficacy in binding to GNPs, reinforcing membrane rigidity, and inhibiting Gyr-B.

In addition to gold nanocomposites, porous CuO nanobastons are another class of nanometric structures, characterized by their rod-like shape and porous surface [113]. In this context, Mansi et al. [114] investigated the fabrication of microwave-induced porous CuO nanobastons loaded with quercetin and rutin. Metal nanoparticles, including CuO, have been extensively studied due to their antimicrobial properties and their diverse physical, biological, and chemical effects [115,116].

Several studies have highlighted the antimicrobial efficacy of silver nanoparticles (AgNPs) when combined with flavonoids such as quercetin. Hooda et al. [69] synthesized quercetin-impregnated silver nanoparticles, which exhibited enhanced antibacterial activity against *K. pneumoniae* ATCC 790603 as assessed by the agar well diffusion method. This increased activity can be attributed to the cell lysis mechanism inherent to silver nanoparticles.

Additionally, the therapeutic potential of gold nanoparticles (AuNPs) has been well-documented, owing to their remarkable properties and straightforward production methods [8]. Khosravi et al. [80] investigated AuNPs synthesized via green methods and coated with *Anthemis atropatana* extract against the resistant strain *K. pneumoniae* ATCC 13883. These AuNPs demonstrated antibacterial activity through mechanisms including increased intracellular oxidative stress mediated by reactive oxygen species (ROS), damage to the bacterial cell membrane resulting in altered permeability, and enhanced internalization of AuNPs. Furthermore, AuNPs exhibited efflux pump inhibitory activity, reduced exopolysaccharide production, urease inhibition, and low cytotoxicity. They also significantly decreased the expression of the *mrkA*, *wzm*, and *acrB* genes [117,118]. This reduction may be attributed to the inhibition of bacterial gene transcription by ROS and/or direct interaction of AuNPs with transcription factors involved in the regulation of these gene expressions.

In the study by Ali et al. [77], magnetic iron oxide nanoparticles were used to encapsulate epigallocatechin gallate (EGCG). The authors concluded that the observed antibacterial activity against resistant clinical isolates of *K. pneumoniae*, as evaluated by the microdilution method, and the anti-biofilm efficacy, assessed using the crystal violet method, were attributable to the ability of EGCG-MINPs to facilitate the penetration of EGCG through the bacterial cell wall and biofilms. This effect was further enhanced by the release of Fe^3+^ ions from the biomolecules, suggesting a potential interference with quorum sensing. Additionally, in silico molecular docking analyses demonstrated that EGCG-MINPs or Fe-EGCG complexes exhibited significantly higher binding affinity for human serum albumin (HSA) compared to free EGCG and Fe^3+^. These findings suggest that EGCG-MINPs could be promising nanosystems for the delivery of biocompatible nanodrugs in in vivo drug delivery applications.

### 3.4. Other Nanostructures

Carbon nanostructures have emerged as a significant innovation within the scientific community, attributed to their capacity to integrate unique properties with the inherent versatility of nanostructures, thereby unlocking novel avenues for advanced applications [41,102]. In this context, extensive research has been conducted on the delivery of quercetin, exemplified by the work of Shabana et al. [66], who encapsulated this flavonoid within mesoporous silica nanoparticles (QMSN) and subsequently assessed its antimicrobial efficacy via the disk diffusion method against multidrug-resistant clinical isolates of *Klebsiella pneumoniae*. The study demonstrated that QMSN at a concentration of 100 μg/mL exhibited a significant antibacterial effect, evidenced by an inhibition zone measuring 10.53 mm, which was notably larger than that observed for free quercetin at the same concentration, where the inhibition zone measured 7.63 mm, as reported in a prior study by Kumar et al. [21]. Furthermore, QMSN has been validated as an effective delivery nanosystem, characterized by its safety in application within living cells or organisms.

In a subsequent investigation, Shameem et al. [67] expanded on these findings by evaluating the impact of QMSNs on growth rates, feed utilization efficiency, health status indicators through biochemical indices, and pathogenic bacterial loads in *Oreochromis niloticus* fry. This study highlighted a notable enhancement in host immunity and resistance to infection by clinical isolates of *Klebsiella pneumoniae*, as assessed through pathogenicity tests, wherein 100 µL of bacterial suspension was injected intraperitoneally into the fish, with daily monitoring over a 15-day period to record mortality rates. This evidence underscores the potential of utilizing natural products, such as encapsulated flavonoids, as viable therapeutic alternatives [72,119].

Another nanotechnological strategy for flavonoid delivery is the employment of nanocrystals—organic nanostructures composed of crystalline particles at the nanometric scale—which serve to provide a protective barrier against degradation, enhance the solubility of bioactive compounds, and facilitate controlled release of therapeutic agents [120]. Memar et al. [74] synthesized rutin-containing nanocrystals (NRs) and evaluated their antibacterial activity using the broth microdilution method, revealing that while free rutin did not demonstrate significant antibacterial activity against environmental strains of *Klebsiella pneumoniae*, NRs exhibited markedly enhanced antibacterial properties.

Further advancements in the field were achieved by Seetharaman et al. [73], who developed nanocomposites of reduced graphene oxide conjugated with gold nanoparticles and hesperetin-coated flavonoids (Hes-Au/rGONCs), assessing their antimicrobial activity via the disk diffusion method. Their results indicated that Hes-Au/rGONCs significantly increased the bioavailability of hesperetin, induced cell death through the generation of reactive oxygen species (ROS), and demonstrated high antibacterial activity against *Klebsiella pneumoniae* MTCC-530. Collectively, these studies illustrate that the use of nanocarriers for flavonoid encapsulation represents a promising strategy to enhance their therapeutic efficacy. By improving stability, solubility, and the ability to traverse biological barriers, nanocarriers facilitate the application of these compounds in treating bacterial infections caused by *Klebsiella pneumoniae*.

## 4. Efficacy and Toxicity of Nanostructures

The nanosystems discussed in this review exhibit a wide range of characteristics and applications, each offering distinct advantages depending on the therapeutic need. Thus, the selection of the appropriate nanocarrier should consider the desired route of administration, the need for controlled release, and the specific properties required for the particular application.

Nanoemulsions are effective for both oral and topical administration, improving the solubility and bioavailability of compounds. In contrast, liposomes, which are ideal for intravenous or topical administration due to their ability to encapsulate lipophilic drugs, are ineffective for oral use due to degradation by gastrointestinal lipases [121,122,123]. Nanohydrogels offer remarkable flexibility in controlled drug release, with bacterial cellulose hydrogels standing out as advanced systems for drug delivery and biomedical applications [96]. Nanofibers, in turn, are widely used in tissue engineering and sustained release systems due to their high surface area and ability to mimic the extracellular matrix [124,125].

Polymeric micelles and nanocrystals are efficient in solubilizing poorly soluble drugs. Unlike liposomes, micelles, with their hydrophobic core and hydrophilic shell, offer greater stability and protection for drugs in aqueous environments and adverse gastrointestinal conditions [126,127,128]. PLGA nanoparticles and nanospheres are widely recognized for their biocompatibility and effectiveness in controlling drug release, making them suitable for various therapeutic applications [129,130].

Chitosan nanoparticles are valued for their biodegradability and potential for oral, intranasal, and topical administration, while mesoporous silica nanoparticles (MSNs) offer high loading capacity and controlled release, making them ideal for targeted therapies [131,132,133]. Gold compounds and silver nanoparticles have antimicrobial properties, used in topical treatments and disinfection, with gold compounds also showing potential in cancer therapies [64,109]. Magnetic iron oxide is used in imaging and targeted therapies due to its magnetic properties, and reduced graphene oxide nanocomposites with gold nanoparticles combine electronic and magnetic properties, showing promising applications in sensors and therapies [134].

The synergy between nanocarriers and therapeutic agents is a crucial aspect of advancing nanotechnology-based therapies, particularly in enhancing antimicrobial activity. A notable example of this synergy is the combination of silver nanoparticles, which have well-established antimicrobial properties, with bioactive compounds such as flavonoids. Similarly, chitosan, a natural polymer with antimicrobial properties, can create release systems that combine its antimicrobial actions when used as a carrier. This synergistic effect between nanocarriers and flavonoids results in a more effective therapeutic approach, amplifying antimicrobial activity [83,109,135].

Although there are no in vivo studies on the use of flavonoid-containing nanoparticles for treating pulmonary infections caused by *Klebsiella pneumoniae*, there are studies that explore the applications of flavonoid-containing nanoparticles for intranasal administration. These findings suggest that such a nanotechnological approach may offer new opportunities for effective delivery and enhancement of treatment for infections caused by *K. pneumoniae* [136].

Encapsulating flavonoids in nanometric structures can enhance their stability, solubility, and ability to cross biological barriers. This approach often allows for the use of lower concentrations, which in turn reduces the toxicity of therapeutic regimens [98,137].

Riaz et al. [138] conducted a study to evaluate the antibacterial activity and in vivo hemolysis rate of conjugated flavonoids (CFs) and gold nanoparticles coated with flavonoids (FAuNPs). The results showed that both CF and FAuNPs exhibited antimicrobial activity against Gram-positive and Gram-negative bacteria. The encapsulation of flavonoids led to a significant reduction in the minimum inhibitory concentration (MIC), decreasing from 500 µg/mL to 25 µg/mL. Furthermore, in the hemolysis experiment, the nanoparticles demonstrated a significantly lower hemolysis rate compared to free flavonoids. At a concentration of 150 µg/mL, FAuNPs exhibited only half the hemolysis rate observed with free flavonoids, which had a hemolysis rate of approximately 7%. These findings highlight the potential of gold nanoparticles coated with flavonoids as a promising approach to reduce toxicity and enhance the therapeutic efficacy of these compounds.

In a study by Elkhateeb et al. [139], the antimicrobial activity of curcumin (CUR) and curcumin-loaded nanostructured lipid carriers (CURC-NLCs) was evaluated against Gram-negative and Gram-positive bacteria. The results revealed that CUR demonstrated antibacterial activity with an MIC ranging from 31.25 to 500 µg/mL. In contrast, CURC-NLCs exhibited lower MICs, ranging from 15.6 to 250 µg/mL, indicating reduced toxicity and increased safety of the compound when encapsulated.

The use of carbon nanotubes incorporated with flavonoids is also a well-known and elucidated nanosystem. When incorporating these compounds, assessing cytotoxicity is crucial. In a study, Espíndola et al. [140] evaluated single-walled and multi-walled carbon nanotubes as nanocarriers for the delivery of 7-hydroxyflavone (7-HF) and assessed their cytotoxic effects on several cell lines, including Vero E6 (normal monkey kidney epithelial cells). The nanotubes exhibited nearly negligible cytotoxicity, maintaining cellular viability, which demonstrates the safety and reliability of these nanosystems.

Shabana et al. [88] conducted a study on mesoporous silica nanoparticles loaded with quercetin against bacterial infections such as *Klebsiella pneumoniae* in *Oreochromis niloticus* species. In addition to antimicrobial evaluation, cytotoxicity was assessed in the tilapia gill cell line (TG). The results showed a cell viability rate above 90% across five different concentrations ranging from 20 to 100 µg/mL. These findings suggest that these nanoparticles have promising potential as therapeutic agents for treating bacterial infections in aquaculture. However, future in vivo studies and evaluation of these nanoparticles’ effects in pulmonary models are essential, considering that *Klebsiella pneumoniae* is a significant pathogen in pulmonary diseases. This would enhance understanding of their efficacy and safety in clinical contexts.

## 5. Material and Methods

The methodology employed in this narrative review involved a comprehensive screening of the literature to identify original studies that provided information on nanocarriers for the delivery of flavonoids against *K. pneumoniae*. Specifically, independent searches were conducted for articles published from 2019 to June 2024 in the PubMed, Science Direct, Google Scholar, and Scopus databases using the following search terms: “*K. pneumoniae* AND nanostructure AND delivery AND flavonoids”. Excluded from the review were reviews, systematic reviews, letters to the editor, and other non-original studies. Additionally, studies written in languages other than English, those without access to the full text, studies with aggregated data, and those involving animals were excluded. Studies that did not provide detailed information on the analysis of flavonoids in plant extracts were also excluded.

## 6. Conclusions

The synthesis of nanocarriers for flavonoid delivery has gained substantial recognition as a highly advantageous strategy, heralding a new therapeutic paradigm aimed at combating both sensitive and resistant strains of *Klebsiella pneumoniae*. The application of nanotechnology in this context optimizes the bioavailability of flavonoids, thereby significantly enhancing their delivery efficacy and amplifying their therapeutic effects against *K. pneumoniae*.

Among the most prevalent flavonoids isolated from plants, quercetin, rutin, catechins, kaempferol, and apigenin are particularly noteworthy. However, their clinical efficacy is often compromised by low water solubility and susceptibility to degradation. To address these limitations, flavonoids and their derivatives can be encapsulated and/or delivered using various nanocarriers, including mesoporous silica nanoparticles, nanohydrogels, silver nanoparticles, gold nanocomposites, CuO nanorods, poly(lactic-co-glycolic acid) nanoparticles, graphene oxide nanocomposites, nanocrystals, methoxy poly(ethylene glycol)-poly(lactide) micelles, magnetic iron oxide nanoparticles, nanoemulsions, polymeric nanoparticles, liposomes, and nanofibers. This encapsulation strategy is promising as it protects flavonoids from adverse environmental factors, enhances their solubility, and enables controlled release and targeted delivery, thereby expanding their therapeutic potential.

In this context, it is imperative to explore and optimize different controlled-release systems and to tailor nanocarriers to address the specific characteristics of infections and resistance profiles associated with *Klebsiella pneumoniae*. Such advancements are crucial, as the discussed nanocarriers have the potential to establish themselves as new therapeutic modalities based on nanotechnology. Additionally, it is essential to conduct further evaluations on the biocompatibility and toxicity of the employed nanomaterials, ensuring that their clinical use does not compromise patient safety. These studies are vital to ensure that the therapeutic benefits of nanocarriers outweigh any potential risks, thereby promoting safe and effective application in clinical practice.

The application of nanostructures for flavonoid delivery represents a significant advancement in the development of novel therapeutic regimens against infections caused by *Klebsiella pneumoniae*. Continued in vivo and clinical studies are therefore essential to develop innovative approaches for directing flavonoids to specific targets, maximizing their antibacterial potential, and overcoming bacterial resistance.

## Figures and Tables

**Table 1:** Physicochemical characteristics of nanostructures for flavonoid delivery.

Nanostructure	Carrier	Flavonoid	Antimicrobial Activity (MIC or IZD)	Size (nm)	Zeta Potential (mV)	PDI	EE %	Reference
Lipid	Nanoemulsion	Catechins	-NE I: P60: 5.5 mg/mL CB: 15 mg/mL -NE II: P60: 8 mg/mL Cur: 2 mg/mL	58 (P60 + Cran NE) 211.2 (P60 + Cur NE)	−16 (P60 + Cran NE) −32.7 (P60 + Cur NE)	ND	ND	Kaur et al. [75]
	Liposomes	Extracts rich in flavonoids	31.25 mg/mL	365.22 ± 0.70	+39.30 ± 0.61	0.351	81.5	Rubaka et al. [76]
Polymeric	Nanohydrogel	Quercetin	500 mg/mL	743.6	+12.1	0.507	ND	Abbaszade et al. [77]
	Nanofibers	Extracts rich in flavonoids	24 mm and 25 mm	50 to 200	ND	ND	ND	Kannan et al. [78]
	Micelles	Luteolin	ND	ND	ND	ND	ND	Miao et al. [79]
	Poly(lactic-co-glycolic acid) nanoparticles) (PLGA);	Hesperidin	100 µg/mL	ND	ND	ND	ND	Balakrishnan et al. [80]
	PLGA nanocapsules	Epigallocatechin gallate (EGCG)	16 µg/mL	61.37 ± 5.90	–11.83 ± 3.22	0.125 ± 0.027	85.79 ± 5.89	Alotaibi et al. [81]
	Nanoemulsion	Essential oil rich in flavonoids	22 mm	ND	ND	ND	ND	Zhang et al. [70]
	Chitosan nanoparticles	Extracts rich in flavonoids	4.1 µg/mL	349.6	ND	ND	ND	Qanash et al. [82]
	Chitosan nanoparticles	Extracts rich in flavonoids	625 μg/mL.	461.7 (NLC-Ginger) 476.7 (NLC Rosemary) 22.04 (CS-Ginger) 45.77 (CS Rosemary)	−25.9 (NLC-Ginger) −32.7 (NLC-Rosemary) +0.144 (CS-Ginger) +0.270 (CS-Rosemary)	0.356 (NLC-Ginger) 0.45 (NLC-Rosemary) 0.261 (CS-Ginger) 0.984 (CS-Rosemary)	92.53 (NLC-Gengibre); 93.47 (NLC Rosemary) 98.91 (CS- Ginger) 94.51 (CS-Rosemary)	Abozahra et al. [83]
Metallic	Gold nanocomposites	Quercetin, Kaempferol, and chrysin	60 μg/mL	4.10 ± 2 to 35 ± 2	ND	ND	ND	Alhadrami et al. [84]
	Silver nanoparticles	Quercetin	ND	48.37	ND	ND	ND	Hooda et al. [85]
	Gold nanoparticles	Extracts rich in flavonoids	6.25–50 µg/mL	22.04 to 45.77	–11.6	ND	ND	Khosravi et al. [86]
	Ferro magnetic oxide nanoparticles	Epigallocatechin gallate	1000 µg/mL	500.6 nm to 1062 nm (diferents pHs)	−21.56	0.299–0.527	ND	Ali et al. [87]
Other	Mesoporous silica nanoparticles (MSN)	Quercetin	100 μg/mL	67.4	51.6	ND	69.1	Shabana et al. [88]
	Mesoporous silica nanoparticles (MSN)	Quercetin	ND	ND	ND	ND	ND	Shameem et al. [89]
	Nanocrystals	Rutin	ND	75 ± 0.16	ND	ND	ND	Memar et al. [90]
	Graphene oxide nanocomposites reduced by gold nanoparticles	Hesperidin	100 µg/mL	21.37 ± 5.7	−36.44	ND	ND	Seetharaman et al. [91]

ND: Not determined.
